# Identification of Salivary Microbiota and Its Association With Host Inflammatory Mediators in Periodontitis

**DOI:** 10.3389/fcimb.2019.00216

**Published:** 2019-06-21

**Authors:** Anna Lundmark, Yue O. O. Hu, Mikael Huss, Gunnar Johannsen, Anders F. Andersson, Tülay Yucel-Lindberg

**Affiliations:** ^1^Division of Periodontology, Department of Dental Medicine, Karolinska Institutet, Huddinge, Sweden; ^2^Science for Life Laboratory, Department of Gene Technology, KTH Royal Institute of Technology, Stockholm, Sweden; ^3^Department of Microbiology, Tumor and Cell Biology, Centre for Translational Microbiome Research (CTMR), Karolinska Institutet, Stockholm, Sweden; ^4^Department of Biochemistry and Biophysics, National Bioinformatics Infrastructure Sweden, Science for Life Laboratory, Stockholm University, Solna, Sweden

**Keywords:** 16S rRNA sequencing, cytokines, inflammatory mediators, microbiome, microbiota, periodontitis, saliva

## Abstract

Periodontitis is a microbial-induced chronic inflammatory disease, which may not only result in tooth loss, but can also contribute to the development of various systemic diseases. The transition from healthy to diseased periodontium depends on microbial dysbiosis and impaired host immune response. Although periodontitis is a common disease as well as associated with various systemic inflammatory conditions, the taxonomic profiling of the salivary microbiota in periodontitis and its association with host immune and inflammatory mediators has not been reported. Therefore, the aim of this study was to identify key pathogens and their potential interaction with the host's inflammatory mediators in saliva samples for periodontitis risk assessment. The microbial 16S rRNA gene sequencing and the levels of inflammatory mediators were performed in saliva samples from patients with chronic periodontitis and periodontally healthy control subjects. The salivary microbial community composition differed significantly between patients with chronic periodontitis and healthy controls. Our analyses identified a number of microbes, including bacteria assigned to *Eubacterium saphenum, Tannerella forsythia, Filifactor alocis, Streptococcus mitis/parasanguinis, Parvimonas micra, Prevotella* sp*., Phocaeicola* sp., and *Fretibacterium* sp. as more abundant in periodontitis, compared to healthy controls. In samples from healthy individuals, we identified *Campylobacter concisus*, and *Veillonella* sp. as more abundant. Integrative analysis of the microbiota and inflammatory mediators/cytokines revealed associations that included positive correlations between the pathogens *Treponema* sp. and *Selenomas* sp. and the cytokines chitinase 3-like 1, sIL-6Rα, sTNF-R1, and gp130/sIL-6Rβ. In addition, a negative correlation was identified between IL-10 and *Filifactor alocis*. Our results reveal distinct and disease-specific patterns of salivary microbial composition between patients with periodontitis and healthy controls, as well as significant correlations between microbiota and host-mediated inflammatory cytokines. The positive correlations between the pathogens *Treponema* sp. and *Selenomas* sp. and the cytokines chitinase 3-like 1, sIL-6Rα, sTNF-R1, and gp130/sIL-6Rβ might have the future potential to serve as a combined bacteria-host salivary biomarker panel for diagnosis of the chronic infectious disease periodontitis. However, further studies are required to determine the capacity of these microbes and inflammatory mediators as a salivary biomarker panel for periodontitis.

## Introduction

Periodontitis is a microbial-induced chronic inflammatory disease affecting tooth-supporting structures. It is highly prevalent, with 46% of the adult population estimated to suffer from periodontitis (Eke et al., 2015). Its severe form, afflicting 10–15% of the population (Eke et al., 2012; Petersen and Ogawa, 2012; Kassebaum et al., 2014), may not only cause tooth loss, but may also lead to an increased risk of systemic diseases such as atherosclerosis, diabetes, and rheumatoid arthritis (Genco and Van Dyke, 2010; Kebschull et al., 2010; Lalla and Papapanou, 2011; Hajishengallis, 2014, 2015; Olsen, 2015).

The pathogenesis of periodontitis is initiated when a biofilm, known as dental plaque, accumulates in proximity to the gingiva. Substances from the biofilm, such as lipopolysaccharides and toxins, activate a host immune response leading to release of various inflammatory mediators (such as prostaglandin E_2_) and cytokines such as interleukins (ILs) and tumor necrosis factor α (TNF-α) as well as proteolytic enzymes including matrix metalloproteinases (MMPs) (Page and Kornman, 1997; Darveau, 2010; Yucel-Lindberg and Bage, 2013). Elevated levels of these mediators have been reported in gingival crevicular fluid (GCF) and saliva (Fujita et al., 2012; Ertugrul et al., 2013; Rathnayake et al., 2013) in case of chronic periodontitis, and numerous studies have been performed to investigate their utility as single potential biomarker for periodontitis (Buduneli and Kinane, 2011; de Lima et al., 2016). In clinical practice, the test stick for MMP-8, a key tissue destructive proteinase associated with the progression of periodontitis, has been reported to be available for routine use (Mantyla et al., 2003; Sorsa et al., 2017). However, to our knowledge, there is today no combined host-response associated salivary diagnostic biomarker panel in clinical use for early diagnosis of periodontitis.

It is established that microorganisms are involved in the initiation of periodontitis, but the specific pathogen-host interactions behind the pathogenesis or the immunopathology of the disease are not well-known. In attempts to identify bacteria and their contributions to the development of periodontitis, subgingival dental plaque has been extensively studied. The landmark study by Socransky et al., utilizing DNA hybridization-based methods, identified several bacterial complexes as associated with either health or disease. Among these complexes, the “red complex” consisting of *Porphyromonas gingivalis, Tannerella forsythia*, and *Treponema denticola* was identified as strongly associated with periodontitis (Socransky et al., 1998). However, with the advancements of new sequencing technologies, other species are being recognized and studies have revealed that periodontitis has a polymicrobial etiology and arises from dysbiotic communities that synergistically contribute to inflammation (Hajishengallis, 2015). Previous studies, using 16S rRNA gene sequencing, on plaque samples have reported *Proteobacteria* and *Actinobacteria* phyla as associated with health while the dysbiotic microbial communities associated with periodontitis have a higher representation of bacteria belonging to the phyla *Spirochaetes, Bacteroidetes, Synergistetes*, and *Firmicutes* (Griffen et al., 2012; Abusleme et al., 2013; Ge et al., 2013; Li et al., 2014). In a systematic review by Pérez-Chaparro et al., based on 41 eligible studies, the phyla *Bacteroidetes, Candidatus Saccharibacteria, Firmicutes, Proteobacteria, Spirochaetes*, and *Synergistetes* were found to be associated with periodontitis (Perez-Chaparro et al., 2014).

In addition to studies performed on subgingival dental plaque samples, few studies have also reported on the microbial composition in periodontitis and health using saliva samples, which are more suitable as diagnostic specimen because they are more easily and non-invasively obtained. Using microarray technique (Belstrom et al., 2014), reported that the salivary microbiota in patients with periodontitis differs from that in healthy controls. Their findings included a higher representation of eight bacterial taxa in samples from periodontitis patients, including *Parvimonas micra* and *Filifactor alocis* (Belstrom et al., 2014). Using the more sensitive 16S ribosomal RNA (rRNA) gene sequencing approach (Chen et al., 2015) identified a group of species, centered around *Filifactor alocis*, as associated with periodontitis. Additionally, Takeshita et al. (2016) demonstrated that oral conditions such as decayed teeth, present teeth, periodontal pockets, gingival bleeding, and oral hygiene significantly associated with the phylogenetic diversity of the salivary bacterial populations in a large-scale population-based Japanese study. Moreover, recently it was reported that microbiota is distinct in saliva and subgingival plaque samples, in periodontal health and disease (Chen et al., 2018), and that the salivary microbial composition in periodontitis is reflected by the well-known periodontal pathogens present in subgingival plaque (Yoshizawa et al., 2013; Belstrom et al., 2018). For example, the periopathogens *Porphyromonas gingivalis, Treponema denticola, Prevotella intermedia, Filifactor alocis, Tannerella forsythia*, and *Parvimonas micra* were detected both in site-specific plaque and saliva samples (Belstrom et al., 2017b). Similarly, a significant positive correlation was detected between salivary and subgingival plaque samples in subjects with periodontitis with regard to periopathogens such as *Porphyromonas gingivalis, Treponema denticola, Aggregatibacter actinomycetemcomitans, Tannerella forsythia*, and *Parvimonas micra* (Haririan et al., 2014). Moreover, the abundance of subgingival plaque-specific bacteria in the salivary microbiota was reflected by the severity of the periodontal condition in patients with periodontitis, emphasizing the importance of saliva as diagnostic fluid.

Most of the previous studies have focused on identifying either microbial or host inflammatory mediators as biomarkers for periodontitis and to our knowledge there are limited studies investigating the correlations between the salivary microbiota and inflammatory mediators as biomarker panel for periodontitis. Accordingly, the aim of the current study was to characterize the taxonomic profiles of saliva samples from patients with periodontitis, relative to healthy controls, using high-throughput 16S rRNA gene sequencing. Furthermore, we also investigated the association between the salivary microbiota and host-derived inflammatory mediators in patients with periodontitis and periodontally healthy controls in order to increase knowledge on potential combined salivary diagnostic biomarker candidates for periodontitis.

## Materials and Methods

### Study Population

The study population consisted of 114 participants, 66 patients diagnosed with chronic periodontitis recruited from a periodontal clinic and 48 healthy individuals without periodontitis (all from Stockholm, Sweden). The mean age (and range) for the patients with periodontitis was 60.9 (22–88) years, and for the healthy control group 42.7 (24–67) years. The ratio females/males was 36/30 in the periodontitis group and 32/16 in the healthy control group without any signs of periodontitis. The clinical diagnosis of periodontitis was based on bleeding on probing >30%, tooth sites with probing depth ≥6 mm, clinical attachment level ≥5 mm and radiographic examinations. Individuals in the healthy control group showed no signs of periodontitis, defined as sites with no gingival/periodontal inflammation, probing depth ≤ 3.0 mm, and no bleeding on probing. Regarding systemic diseases in the periodontitis group, one individual had rheumatoid arthritis, three individuals had diabetes, and nine individuals suffered from high blood pressure and two from heart disease. In the healthy control group, no individuals had rheumatoid arthritis or diabetes, but one individual suffered from heart disease. The study was performed in accordance with the Declaration of Helsinki and current Swedish legislation. The sample collection was approved by the Regional Ethics Board in Stockholm with reference number 2014/1588 – 32/3 and written informed consent was obtained from all participants.

### Collection and Preparation of Saliva Samples

Saliva samples were collected from all 114 participants after dental clinical examination. All participants fasted 2 h prior to the examination. Stimulated saliva samples were obtained by chewing paraffin tablets for 2 min whilst the saliva was collected into sterile 50 mL falcon tubes, which were immediately frozen at −20°C. Each vial was thereafter thawed and centrifuged at 500 × g for 10 min and the supernatants were aliquoted into 1.5 ml eppendorf tubes and stored at −80°C until further processing.

### DNA Extraction, 16S rRNA Gene Library Preparation and Sequencing

For 16S rRNA gene analysis, 48 samples from patients with periodontitis and 48 samples from healthy controls were included. This cohort size achieved a power of at least 0.80 to detect a difference in the taxonomic composition, based on an estimated read count of at least 10,000. Power calculations were based on the Dirichlet-multinomial distribution as described previously (Padovani et al., 1986). Prior to DNA extraction, 400 μl saliva was centrifuged at 10,000 rpm for 15 min. The supernatants were removed and the pellet resuspended in 200 μl phosphate buffered saline (PBS). DNA was thereafter extracted from the suspensions using QIAamp DNA Mini Kit (Qiagen, Valencia, CA, USA) and eluted into 50 μl H_2_O. The DNA concentrations were measured using Qubit® 2.0*Fluorometer*(*Invitrogen, Carlsbad, CA, USA*).*Thereafter, theV*3−−*V*4*regionsofthebacterial*16*SrRNAgenewereamplifiedwith*1.0μM 341′F primer (CCTAHGGGRBGCAGCAG) and 1.0 μM 805R primer (GACTACHVGGGTATCTAA TCC) (Herlemann et al., 2011), KAPA HotStart ReadyMix (KAPA Biosystems, Wilmington, MA, USA), 0.5 ng/μl bovine serum albumin (New England Biolabs, Ipswich, MA, USA) and 2.0 ng DNA. PCR conditions during the amplification were 98°C for 2 min followed by 26 cycles of 98°C for 20 s, 54°C for 20 s, and 72°C for 15 s, and a final elongation step of 72°C for 2 min. After amplification, the samples were purified with Polyethylene Glycol 6000 (Merck Millipore, Darmstadt, Germany) and carboxylic acid beads (Dynabeads® MyOne™, Thermo Fisher Scientific, Waltham, MA, USA) using the procedure described by Lundin et al. (2010). Indexing was thereafter performed with 12 μl of the amplified and purified product, 0.4 μM forward and 0.4 μM reverse indexing primer and KAPA HotStart ReadyMix (KAPA Biosystems, Wilmington, MA, USA). The PCR cycling conditions were 98°C for 2 min followed by 10 cycles of 98°C for 20 s, 62°C for 30 s, and 72°C for 30 s, and a final elongation step of 72°C for 2 min. After amplification the samples were quantified using Qubit® 2.0*Fluorometer*(*Invitrogen, Carlsbad, CA, USA*), *dilutedto*2.0*ng*/μl and pooled eight by eight before purification by the same procedure as described above. Agilent 2100 Bioanalyzer (Agilent Technologies, Santa Clara, CA, USA) and Qubit® 2.0*Fluorometer*(*Invitrogen, Carlsbad, CA, USA*)*wereusedforcheckingtheampliconfragmentsizesandquantification*.*EquimolaramountsofindexedsamplesweremixedandsequencedwithIlluminaMiSeq*(*IlluminaInc, SanDiego, CA, USA*)*atNGI*/*SciLifeLabStockholm*.

### Sequence Data Processing

The median depth of sequencing was 189,948 reads (IQR = 111,170–299,760) per sample. The samples had <10,000 reads and were excluded from further analyses, which resulted in the inclusion of 46 patients with periodontitis and 47 healthy controls without periodontitis. To retrieve the biological sequences from the reads, we adopted the DADA2 pipeline, which enables inference of biological sequences from amplicon reads by modeling Illumina sequencing errors (Callahan et al., 2016). After trimming and filtering low-quality reads and sequencing artifacts (e.g., primers, PhiX, chimeras, etc.), the retained reads with average Phred score *Q* ≥ 25 were denoised using DADA2. The paired-end reads were then merged by requiring 30 bp overlap with no mismatches. A total of 3,695 amplicon sequence variants (ASVs) were finally inferred from 93 samples (8,531,488 denoised reads) and were then taxonomically annotated with RDP classifier (RDP training set 14 Wang et al., 2007) by requiring a minimum bootstrap threshold of 80. The reads belonging to the inferred ASVs were agglomerated to 17 phyla, 28 classes, 51 orders, 103 families, and 200 genera. We then filtered out low-count taxa by including only ASVs with at least one read in at least 5% of the samples, resulting in the inclusion of 719 ASVs, assigned to 12 phyla, 21 classes, 31 orders, 58 families, and 94 genera. These sequences were further annotated with RDP species-level training set (version 14), and eventually, 483 sequences received species assignments. The reason that only approximately half of the ASVs got species assignment is due to the fact that only the V3–V4 region of the 16S gene was sequenced. However, the 16S primer set used in this study has a well-balanced coverage on all bacterial taxonomies and can generate amplicons of a propriate length for Illumina MiSeq sequencing. A phylogenetic tree was constructed using the scripts align_seqs.py, filter_alignment.py and make_phylogeny.py provided by QIIME (Caporaso et al., 2010). The resulting data was used in all downstream analyses as described below.

### Analysis of Sequence Data

All statistical analyses were conducted in R (version 3.3.0) (R Core Team, 2015). The DADA2 table, sample data, and taxonomic table were combined into a phyloseq object using R library phyloseq (McMurdie and Holmes, 2013). Univariate differential abundance analysis was performed on different taxonomic levels (Phylum, Class, Order, Family, and Genus) as well as on ASVs using DESeq2 (Love et al., 2014). The phylogenetic tree was merged with the phyloseq object and Principal Coordinates Analysis (PCoA) and analysis of similarities (ANOSIM) test were performed on unweighted UniFrac distances with taxa having a coefficient of variation >3.0. Next, random forest classification was performed on the relative abundances of ASVs using the randomForest package (Liaw and Wiener, 2002) in R. The variable importance scores, corresponding to the mean decrease Gini, were extracted and visualized together with the log2 fold changes of the ASVs.

### Determination of Cytokine and Inflammatory Mediator Levels in Saliva Samples

A total of 37 different cytokines and inflammatory mediators were analyzed in 111 saliva samples (three patients excluded due to not enough saliva volume) using multiplex immunoassay technology on a Bioplex Suspension Array System (Bio-Rad Laboratories, Hercules, CA, USA) with the Bio-Plex Pro Human Inflammation Panel I 37-Plex assay (Bio-Rad Laboratories, Hercules, CA, USA). Cytokine levels that were below each analyte's sensitivity level were substituted with the sensitivity level of each respective analyte. In cases where more than 50% of samples had values below the sensitivity level the cytokine was excluded from further analysis. Comparisons of cytokine levels between samples from periodontitis patients and samples from healthy controls were performed using Mann-Whitney *U*-test. *P* < 0.05 were considered significant and false-discovery rate (FDR) adjusted *P*-values were calculated using Benjamini-Hochberg correction.

### Correlation Analysis Between Microbiota and Inflammatory Mediator Levels

To investigate the correlations between microbiota and various host-mediated inflammatory mediators such as cytokines, a sparse partial least squares discriminant analysis (sPLS-DA) method, implemented in the mixOmics R package (Le Cao et al., 2011; Rohart et al., 2017) was used to perform integration and visualization of microbial and cytokine data. The cytokine data and the relative abundances of the ASV data were normalized to zero mean and unit variance prior to analysis with sPLS-DA.

## Results

### 16S rRNA Gene Amplicon Sequencing

In the current study, we analyzed the microbiota by performing 16S rRNA gene sequencing on saliva samples from 96 patients diagnosed with chronic periodontitis and controls without periodontitis. Samples with <10,000 reads were excluded from analysis, which resulted in the inclusion of a total of 46 patients with periodontitis and 47 healthy controls without periodontitis. The diagnosis of periodontitis was established by means of bleeding on probing >30%, tooth sites with probing depth ≥ 6 mm, clinical attachment level (≥5 mm) and radiographic examinations. Subjects in the control group showed no signs of periodontitis, defined as sites with no gingival/periodontal inflammation, probing depth (≤3.0 mm), and no bleeding on probing. The median depth of sequencing, after removing samples <10,000, was 195,321 reads (IQR = 114,165–300,854) per sample.

### Bacterial Community Composition

**Figure 1:**
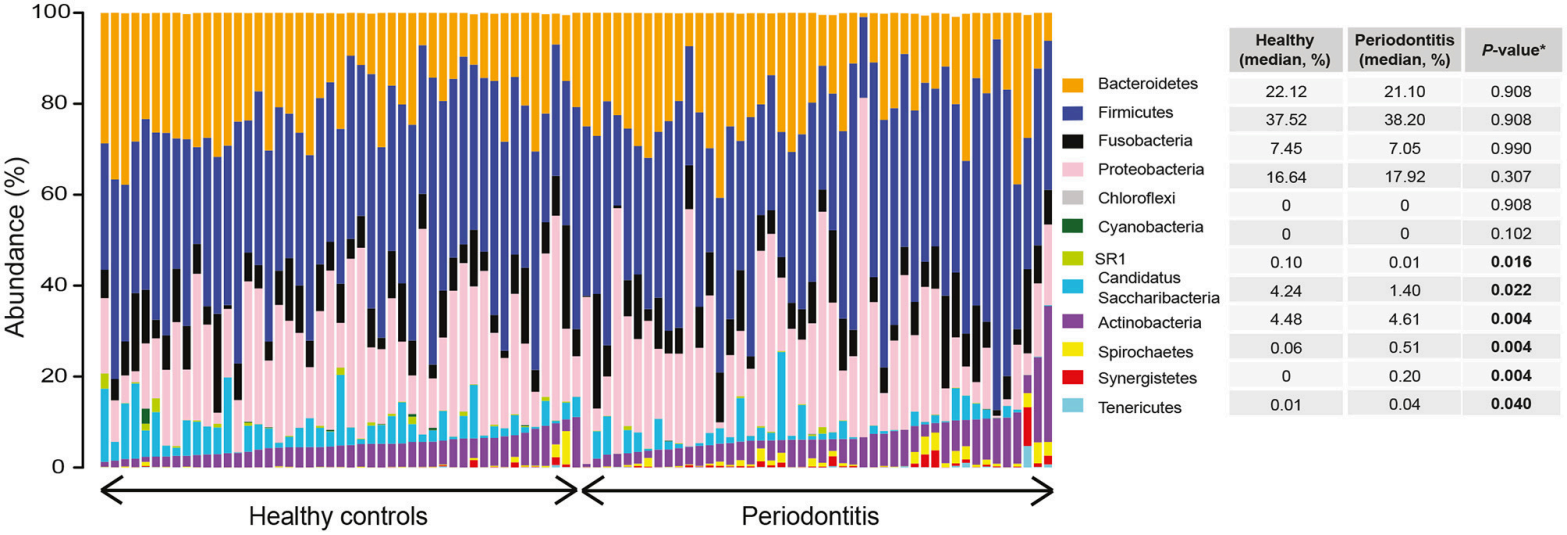
Relative abundance distribution at phylum level of saliva samples from periodontitis patients and healthy controls. The bar plots depict the relative abundance of microbiota at phylum level for all samples, grouped according to their disease status (periodontitis or healthy). The median relative abundance of each phylum in saliva, in samples from patients with periodontitis and healthy controls, are also demonstrated together with the adjusted *P*-value. Significantly (adjusted *P* < 0.05) different levels are indicated with boldface.

A total of 12 phyla, 94 genera, and 719 amplicon sequence variants (ASVs) were detected in the saliva samples of patients with periodontitis and healthy controls, after filtering out lowly abundant ASVs. Relative abundance distribution at phylum level for saliva samples from healthy controls and patients with periodontitis is demonstrated in Figure 1. The most predominant phyla across all samples were *Firmicutes* (median relative abundance in healthy controls and patients with periodontitis 37.5 and 38.2%, respectively), *Bacteroidetes* (22.1 and 21.1%), and *Proteobacteria* (16.6 and 17.9%). The remaining ASVs in the two groups were assigned to the phyla *Fusobacteria, Actinobacteria, Candidatus Saccharibacteria, Spirochaetes*, candidate division SR1, *Synergistetes, Tenericutes, Cyanobacteria*, and *Chloroflexi* (Figure 1).

### Microbiota Ordination and Taxon Distribution

**Figure 2:**
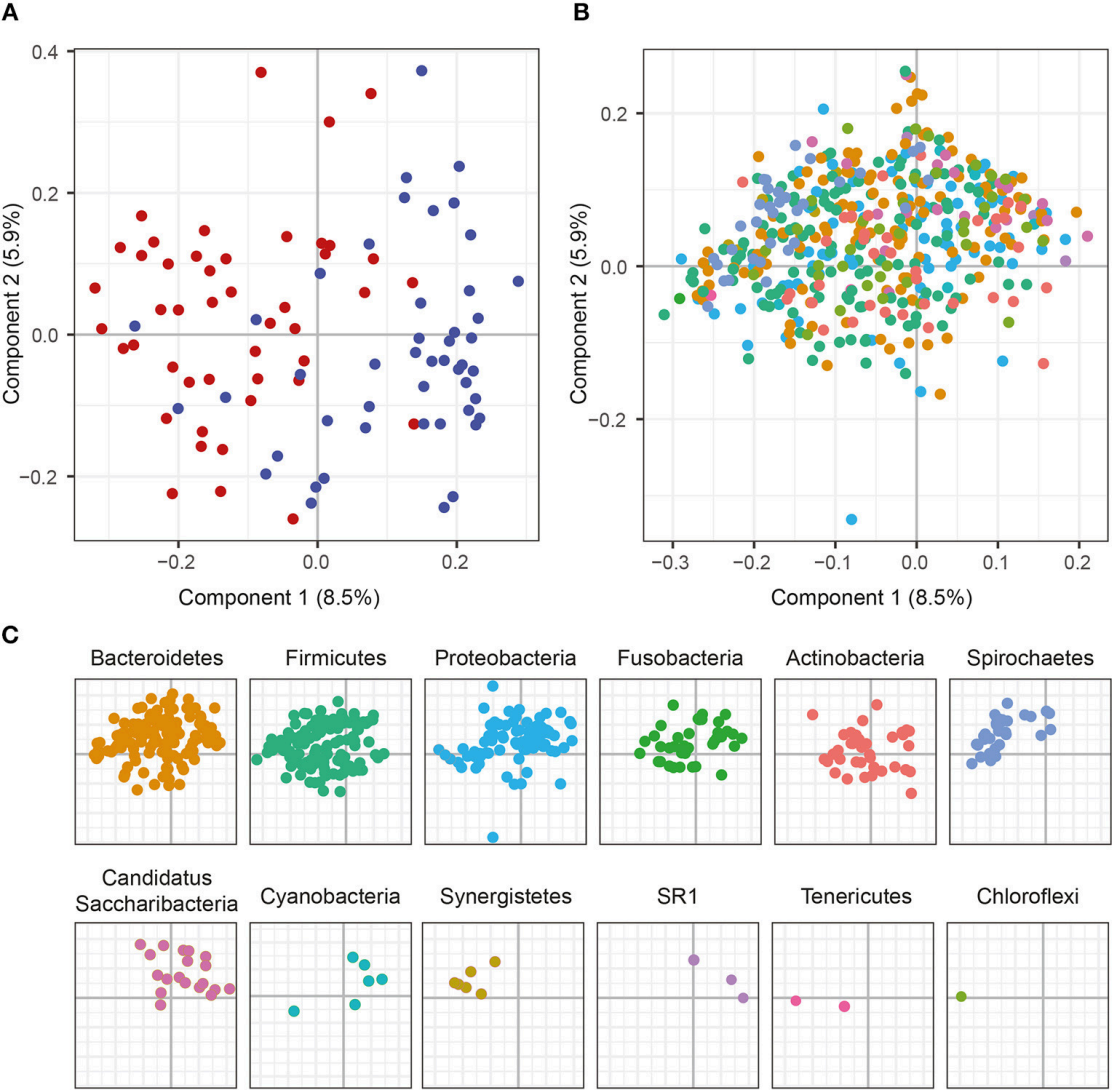
Principal Coordinate Analysis (PCoA) plots on the first two coordinates with UniFrac distances. Variations explained by the coordinates are given within parenthesis. **(A)** A sample projections plot with the samples color-coded by disease status. Saliva samples from patients with periodontitis are highlighted in red and healthy controls are highlighted in blue. **(B)** The contributions of individual sequence variants (ASVs) are visualized in the same PCoA space as the samples and shaded according to their phylum assignment. **(C)** The contributions of the ASVs to the PCoA, split by phyla.

To characterize similarities or differences in the composition of bacterial communities, we next applied Principal Coordinate Analysis (PCoA) to UniFrac distances generated for the microbial data. As shown in Figure 2A, the PCoA separated the samples of patients with periodontitis and healthy individuals along the first component, with samples from patients with periodontitis gathered to the left in the plot and the samples from healthy controls to the right. The distinct microbial compositions between the two groups were verified by ANOSIM analysis, demonstrating significant (*P* < 0.001) difference in microbial communities between periodontitis patients and controls.

To further investigate the underlying biology for this separation, the ASVs were instead visualized in the PCoA and shaded by phylum. This revealed that several phyla also separated along the first component. The results demonstrated that *Spirochaetes, Synergistetes, Tenericutes*, and *Firmicutes* were more abundant to the left in the plot and therefore were more prevalent in samples from periodontitis patients, which also clustered to the left (Figures 2B,C). On the other hand, *Candidatus Saccharibacteria, Cyanobacteria*, and candidate division SR1 clustered to the right in the plot, in the same area as the healthy control samples (Figures 2B,C).

### Differentially Abundant Taxa in Saliva Samples of Patients With Periodontitis and Controls

To identify differences in taxa abundances of saliva samples from patients with periodontitis and healthy controls we used differential expression analysis for sequence count data version 2 (DESeq2) (Love et al., 2014), Differences between health- and periodontitis-associated saliva samples were observed at all phylogenetic levels (Supplementary Table S1). At the phylum level, *Actinobacteria, Spirochaetes, Synergistetes*, and *Tenericutes* were identified as significantly (FDR adjusted *P* < 0.05) more abundant in the salivary microbiota of periodontitis patients, whereas *Candidatus Saccharibacteria* and candidate division SR1 were significantly (FDR adjusted *P* < 0.05) enriched in samples from healthy individuals (Figure 1).

**Figure 3:**
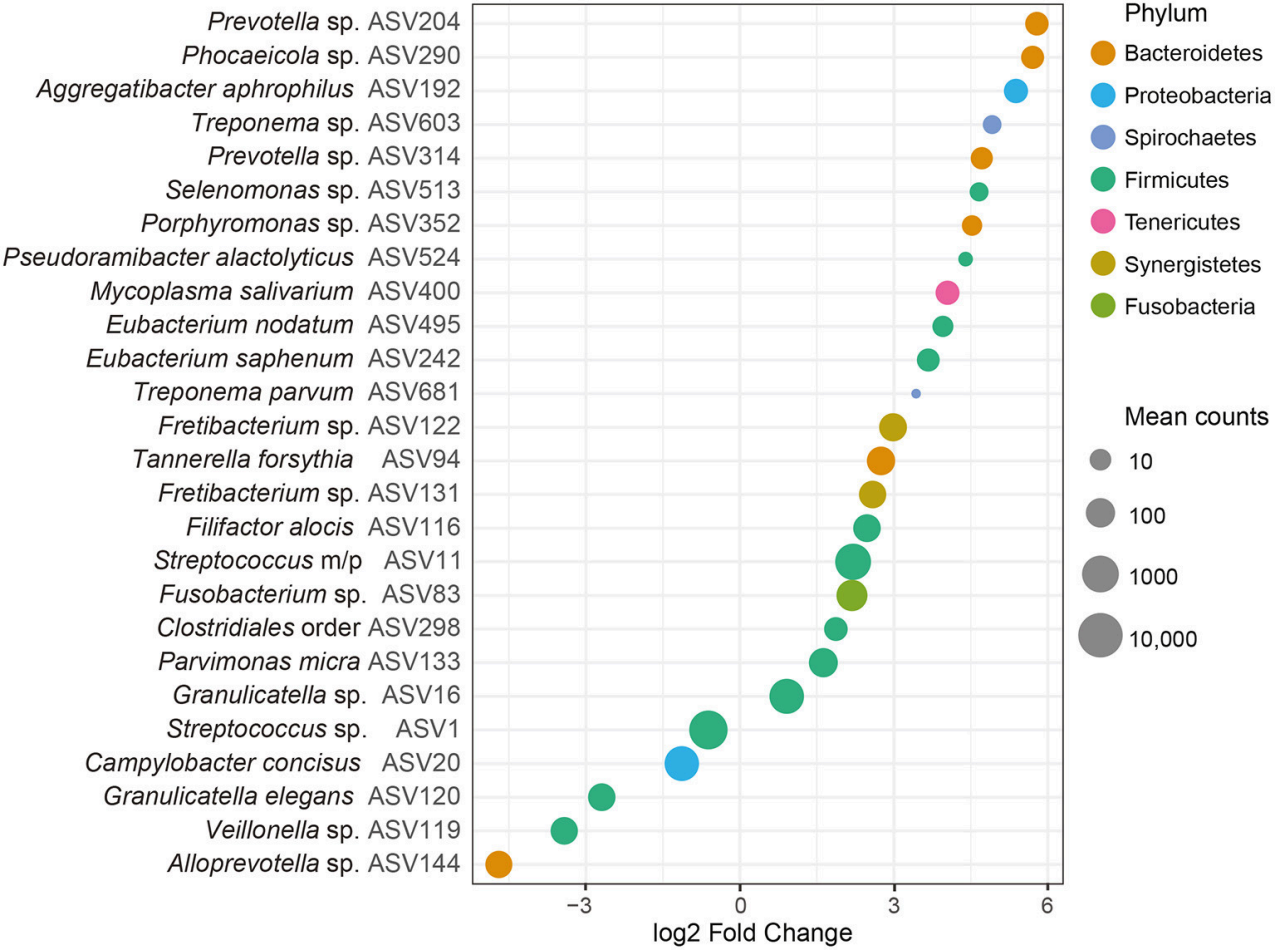
Differentially abundant amplicon sequence variants (ASVs) identified through DESeq2 testing. Each ASV is demonstrated at its lowest annotated taxonomic rank together with its ASV ID. The ASVs are color-coded according to the phyla they belong to and plotted according to their log2 fold change, calculated as the levels in samples from patients with periodontitis relative the levels in samples from healthy controls, with size factor normalization as implemented in DESeq2. Dot sizes correspond to mean counts, after normalization as implemented in DESeq2, and ranges from 3.4 (*Treponema parvum*, ASV681) to 6023.0 (*Streptococcus* sp. ASV1). *Streptococcus mitis/parasanguinis* is shortened to *Streptococcus m/p* (ASV11).

At the ASV level, we identified 26 ASVs that were significantly (FDR adjusted *P* < 0.05) differentially abundant between samples from patients with periodontitis and healthy controls (Figure 3 and Supplementary Table S2). The majority of these ASVs (21 ASVs) were more abundant in our saliva samples from patients with periodontitis, whereas five ASVs were more abundant in samples from healthy controls. The top five ASVs that were more abundant in the samples from periodontitis patients included ASVs assigned to *Prevotella* spp. (ASV204 and ASV314), *Phocaeicola* sp. (ASV290), *Aggregatibacter aphrophilus* (ASV192), and *Treponema* sp. (ASV603). In healthy samples, the top 5 ASVs that were more highly abundant included *Alloprevotella* sp. (ASV144), *Veillonella* sp. (ASV119), *Granulicatella elegans* (ASV120), *Campylobacter concisus* (ASV20), and *Streptococcus* sp. (ASV1) (Figure 3 and Supplementary Table S2).

### Classification of Samples From Patients With Periodontitis and Controls

**Figure 4:**
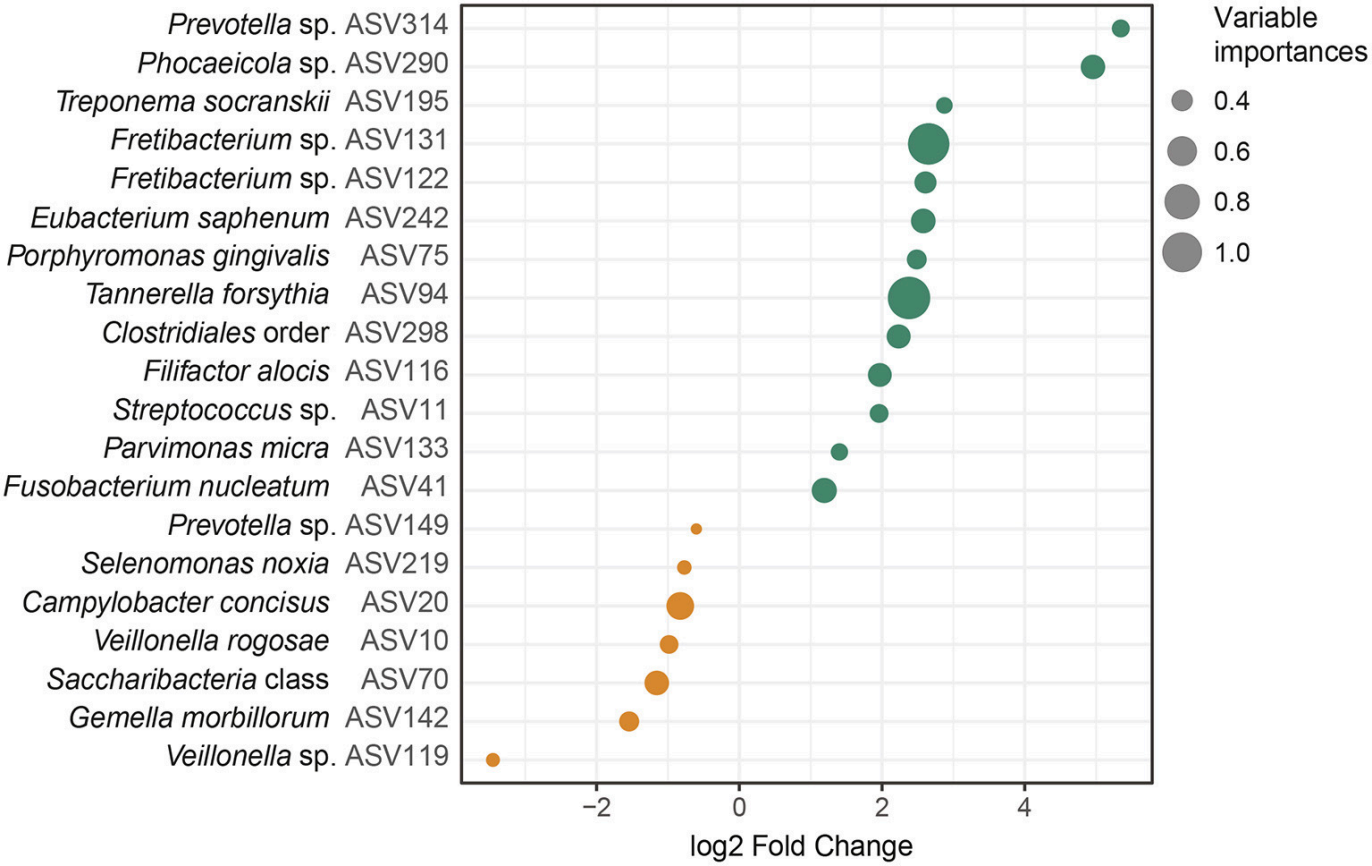
Amplicon sequence variants (ASVs) identified by random forest analysis. The 20 most important ASVs for the classification of saliva samples from patients with periodontitis and healthy controls are included. The variable importances, corresponding to mean decrease Gini are demonstrated together with the log2 Fold Changes, calculated on the relative abundances of the ASVs. The ASVs more abundant in samples from patients with periodontitis are highlighted in green and ASVs more abundant in samples from healthy controls are highlighted in orange. The dot sizes correspond to the variable importances, or mean decrease Gini, with *Tannerella forsythia* having the highest variable importance, 1.14, and *Prevotella* sp. (ASV149) having the lowest variable importance, 0.32. The lowest annotated taxonomic rank is shown for each ASV as well as the IDs of the ASVs used for the analysis. These ASVs showed some variations in fold change compared to the univariate analysis, which was performed with size factor normalization and shrinkage estimation of fold changes, as implemented in DESeq2, while in the random forest model relative abundances were used. *Streptococcus mitis/parasanguinis* is shortened to *Streptococcus m/p* (ASV11).

Next, we tested whether the bacterial abundances observed from the saliva samples could be used to differentiate individuals with periodontitis from those without periodontitis using a random forest model based on the ASVs. This random forest model classified the samples into periodontitis and non-periodontitis subjects with 79.52% accuracy. To investigate the underlying biology for the classification, we extracted the ASVs with the highest importance in the random forest model, and calculated their fold changes. The 20 most important ASVs for the classification of samples are demonstrated in Figure 4 and in Supplementary Table S3. Among the five ASVs with the highest importances, four ASVs were more abundant in samples from periodontitis patients (*Tannerella forsythia* [ASV94], *Fretibacterium* sp. [ASV131], *Fusobacterium nucleatum* [ASV41], and *Eubacterium saphenum* [ASV242]) and one ASV (*Campylobacter concisus* [ASV20]) was more abundant in samples from healthy controls (Figure 4 and Supplementary Table S3).

In agreement with the results from the DESeq2 testing, among the 20 ASVs with the highest importance in the random forest, 12 were also among the significantly differentially abundant ASVs as identified in our DESeq2 analysis. Ten of these ASVs were more abundant in periodontitis samples, whereas two were enriched in samples from healthy controls (Figures 3, 4, and Supplementary Tables S2, S3).

### Inflammatory Mediators in Saliva Samples of Patients With Periodontitis and Controls

**Figure 5:**
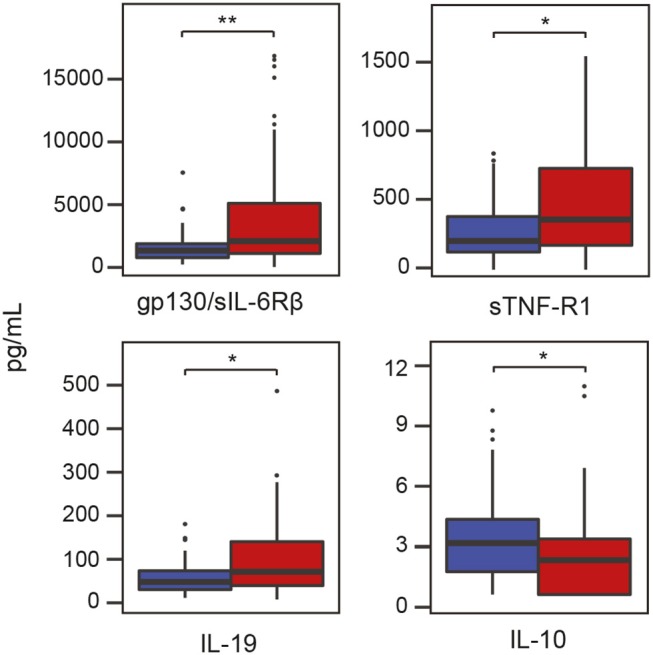
Levels of inflammatory mediators/cytokines in saliva samples from patients with periodontitis and healthy controls. Periodontitis is color-coded in red and healthy controls are color-coded in blue. Gp130/sIL-6Rβ, glycoprotein 130/soluble interleukin-6 receptor β; IL-10, interlukin-10; IL-19, interleukin-19; sTNF-R1, soluble tumor necrosis factor receptor 1; *P < 0.05; **P < 0.05 with Benjamini-Hochberg *post-hoc* test.

In addition to characterizing the salivary microbiota in periodontitis and health, we also measured the levels of various inflammatory cytokines in the same saliva samples using Luminex technology. The results revealed that three cytokines, namely glycoprotein 130 (gp130)/soluble IL-6 receptor β (sIL-6Rβ), IL-19 and soluble TNF receptor 1 (sTNF-R1), were significantly (*P* < 0.05) higher in periodontitis compared to controls (Figure 5). The anti-inflammatory cytokine IL-10, on the other hand, was significantly (*P* < 0.05) lower in samples from periodontitis patients (Figure 5). After FDR adjustments, however, only the inflammatory cytokine gp130/sIL-6Rβ remained significant (FDR adjusted *P* < 0.05).

### Inflammatory Mediators Associated With Microbiota in Saliva

**Figure 6:**
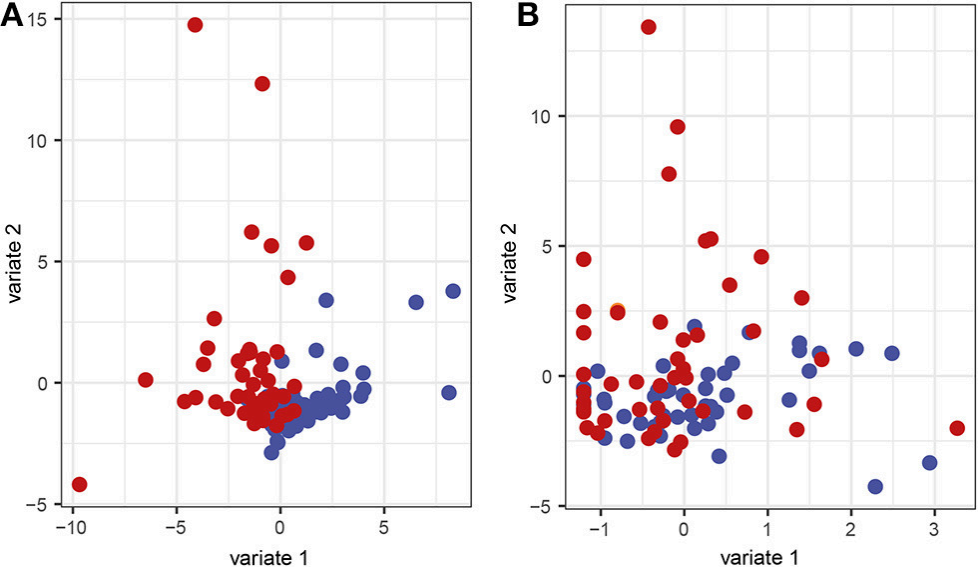
Sparse partial least squares discriminant analysis (sPLS-DA) sample representation. Samples are demonstrated regarding the first and second component based on **(A)** the microbial and **(B)** the cytokine data. Red represents saliva samples from patients with periodontitis and blue represents samples from healthy controls.

In order to investigate interactions between microbiota and corresponding host cytokines in periodontitis, as well as possible combinations of microbiota and host response biomarkers, we used the supervised method *sparse partial least squares discriminant analysis* (sPLS-DA). This classification method contains a selection scheme where only a subset of the ASV frequencies and cytokine abundance levels relevant for separating the classes are retained. The method also generates components, which are linear combinations of the retained features, designed to optimally separate the classes, and which can be used to obtain an informative lower-dimensional representation of the data. In order to visualize the results from the sPLS-DA, sample projections plots of the first and second component with regard to the ASVs as well as the cytokines were created. Separation between samples from patients with periodontitis and samples from healthy controls along the first component could be identified for the ASVs (Figure 6A). However, with regard to the cytokine data, no clear separation could be observed between the two groups (Figure 6B).

**Figure 7:**
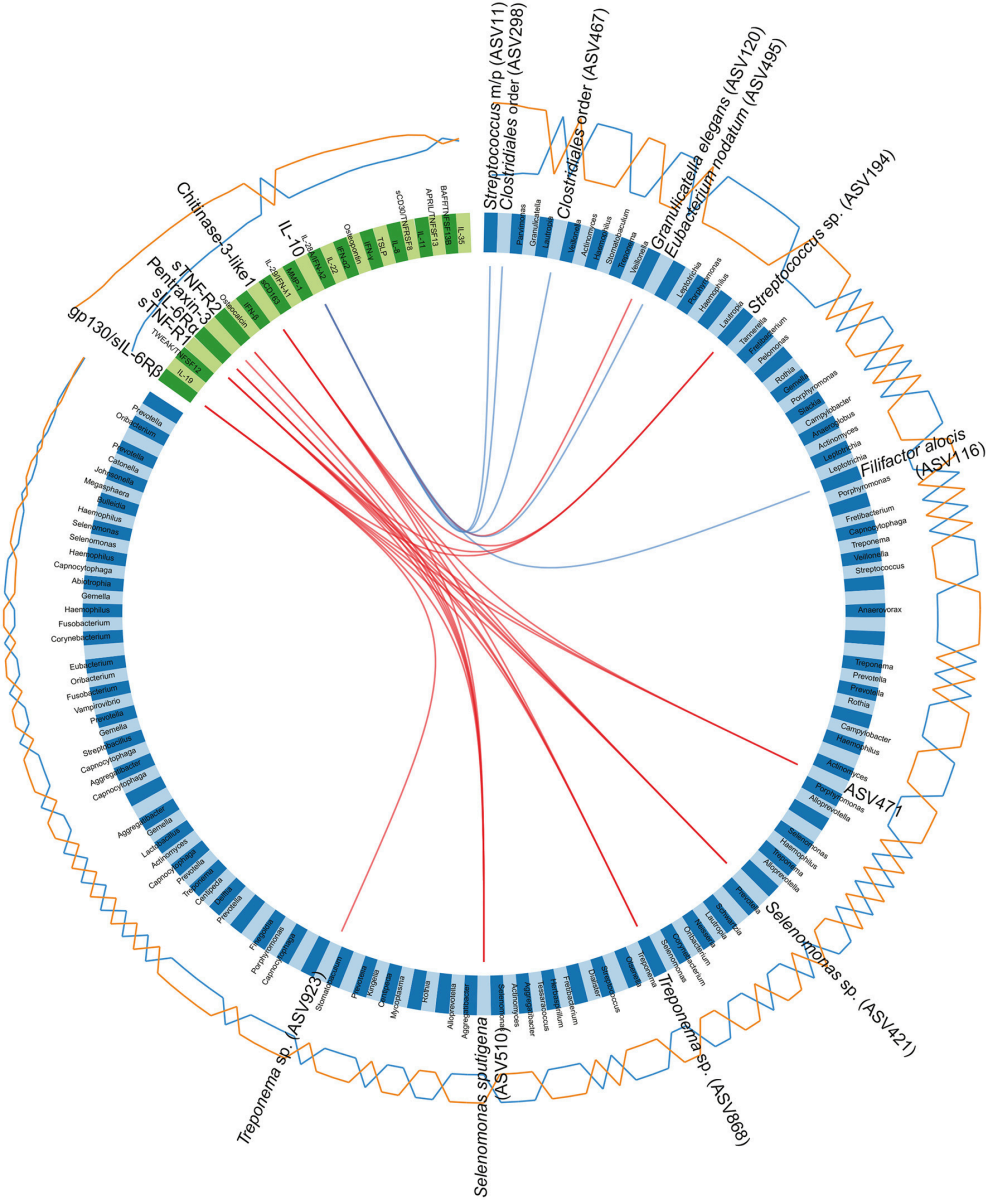
Circos plot depicting correlations between microbiota and cytokines. The panel of amplicon sequence variants (ASVs) and cytokines identified by the sparse partial least squares discriminant analysis (sPLS-DA), regarding the first and second component, are included in the Circos plot. Blue variables represent ASVs and green variables represent cytokines. Orange and blue lines outside the circle represent the abundance of ASVs or the levels of cytokines in samples from patients with periodontitis and healthy controls, respectively. Red and blue lines inside the circle represent positive and negative correlations, respectively, between ASVs and cytokines, at a correlation cutoff of 0.5. The lowest annotated taxonomic rank is shown for each ASV that has a significant correlation with a cytokine, together with its ASV ID used in the analyses. *Streptococcus mitis/parasanguinis* is shortened to *Streptococcus m/p* (ASV11).

The correlations, identified between the ASVs and cytokines through the sPLS-DA, were also visualized in a Circos plot (Figure 7). The plot revealed that, for the first and second component and at a cutoff of 0.5, there was a number of correlations between cytokines and ASVs. The cytokines gp130/sIL-6Rβ, sTNF-R1, sIL6-Rα, pentraxin 3, sTNF-R2, and chitinase 3-like 1 were identified as positively correlated with ASVs belonging to *Streptococcus* sp. (ASV194), *Selenomonas* sp. (ASV421), and *Treponema* spp. (ASV868 and ASV923)*, Selenomonas sputigena* (ASV510) and an unclassified *Bacteria* (ASV471). The anti-inflammatory cytokine IL-10 was identified as negatively correlated to ASVs assigned to *Streptococcus mitis/parasanguinis* (ASV11), *Eubacterium nodatum* (ASV495) and *F. alocis* (ASV116), as well as to two ASVs assigned to the order *Clostridiales* (ASV298 and ASV467). In addition, IL-10 also correlated positively with an ASV assigned to *G. elegans* (ASV120) (Figure 7).

## Discussion

In this study we report specific salivary microbial signatures for periodontitis, as identified by high-throughput 16S rRNA gene sequencing of periodontitis patients and healthy controls. In addition, we report for the first time, to our knowledge, the combined interactions between salivary microbiota and host-mediated inflammatory responses. We identified higher abundance of a number of species in periodontitis, including species previously reported as associated with periodontitis, as well as not yet named species belonging to genera *Prevotella, Phocaeicola*, and *Fretibacterium*, which might be potentially novel species specific for periodontitis. Analysis of the microbiome data in combination with inflammatory mediators, using the same saliva samples, showed positive correlations between *Streptococcus* sp. (ASV194), *Selenomonas* sp. (ASV421), and *Treponema* spp. and the cytokines gp130/sIL-6Rb, sTNF-R1, sIL6-Rα, pentraxin 3, sTNF-R2, and chitinase 3-like 1.

We first investigated the overall composition within the samples at phylum level, which revealed that the phyla *Actinobacteria, Spirochaetes, Synergistetes*, and *Tenericutes* were more abundant in samples from patients with periodontitis while SR1 and *Candidatus Saccharibacteria* were more abundant in samples from healthy controls. Our results regarding *Spirochaetes, Synergistetes*, and *Tenericutes* are consistent with previous findings reported in plaque samples from patients with periodontitis and healthy controls (Griffen et al., 2012; Abusleme et al., 2013; Ge et al., 2013; Li et al., 2014). With regard to SR1, which we identified as more abundant in samples from healthy controls, it has previously been reported at low levels in saliva from healthy individuals (Dewhirst et al., 2010), but, to our knowledge, not been reported as more abundant in health compared to periodontitis. Our findings that *Candidatus Saccharibacteria* (syn. Candidate division TM7) was more abundant in health and that *Actinobacteria* was more abundant in periodontitis are in contrast to previous findings obtained in plaque samples (Liu et al., 2012; Li et al., 2014). However, in line with our results, *Actinobacteria* has been reported as one of the dominating phyla in subgingival microbiota of chronic periodontitis patients (Shi et al., 2018).

In the next series of analyses, DESeq2 (Love et al., 2014) and a random forest machine learning model were employed to identify ASVs that were differentially abundant between saliva from patients with periodontitis and healthy controls. The results from these two methods, largely supporting each other, identified 12 ASVs through both approaches. Among these 12 ASVs, four ASVs could be unambiguously assigned at species level; *E. saphenum, T. forsythia, F. alocis*, and *P. micra*. The pathogen *F. alocis* has previously been identified as more abundant in saliva samples (Belstrom et al., 2014) and has been proposed as the center of a group of pathogens that are associated with periodontitis (Chen et al., 2015). In addition, these four species have previously been associated with periodontitis also in plaque samples (Kumar et al., 2003; Mayanagi et al., 2004; Colombo et al., 2009; Abiko et al., 2010; Griffen et al., 2012; Chen et al., 2015, 2018; Oliveira et al., 2016). Thus, our results demonstrating that these species are also more abundant in saliva samples from periodontitis patients compared to healthy controls, make them promising candidates for further analyses as a salivary biomarker panel for periodontitis. Previous studies reporting distinct oral microbial profiles for health and disease have proposed that bacterial species might be used as potential biomarkers for detection and monitoring different oral diseases (Griffen et al., 2012; Peterson et al., 2013; Belstrom et al., 2014; Kageyama et al., 2017; Chen et al., 2018; Kim et al., 2018). For instance, distinct oral microbial signatures have been associated with failing implants (Kumar et al., 2012), periodontal therapy changes (Chen et al., 2018) and oral cancer (Lim et al., 2018).

The ASVs that could be assigned to genera but not to species included *Prevotella* sp., *Phocaeicola* sp., and *Fretibacterium* sp. as well as one ASV that mapped to both *Streptococcus mitis* and *Streptococcus parasanguinis*. The *S. mitis* organism is a member of the “yellow complex” comprising early colonizers in periodontitis (Socransky et al., 1998) and *Streptococcus parasanguinis* has been reported by Fine et al. (2013) to be involved in localized aggressive periodontitis (Fine et al., 2013). *Prevotella* species, and most frequently *P. intermedia*, have previously been associated with periodontitis (Salminen et al., 2015; Deng et al., 2017). In addition, *Fretibacterium* has over the last years gained attention for its association with periodontitis (Marchesan et al., 2015; Oliveira et al., 2016). *Phocaeicola* has previously been reported as more abundant in subgingival plaque samples of patients with periodontitis compared to healthy controls (Camelo-Castillo et al., 2015; Chen et al., 2018).

With regard to the “red complex,” comprising *P. gingivalis, T. forsythia*, and *T. denticola*, associated with subgingival plaque microbial profiles of periodontitis, two members, *T. forsythia* and *P. gingivalis*, were identified in saliva samples through our 16S rRNA gene analysis. In the random forest model, *T. forsythia* was identified as the most important ASV, which supports this member as highly indicative for periodontitis, whereas *P. gingivalis* was identified as only the twentieth most predictive ASV in our random forest model. In addition, *P. gingivalis* was not identified as significant by our DESeq2 testing. The third member of the red complex, *T. denticola*, did not appear as significant in any of our analyses. Thus, our results verify previous subgingival periodontal pathogens as well as suggest that there might be other group of bacteria which may be more predictive for periodontitis than the “red complex.”

In order to identify associations between cytokines and microbiota as potential diagnostic markers for periodontitis, we next investigated the levels of 37 different cytokines in the same saliva samples. The results showed that the levels of gp130/sIL-6Rβ were significantly higher in samples from patients with periodontitis than in healthy controls. Our novel finding that gp130/sIL-6Rβ is up-regulated in saliva from periodontitis patients is further supported by evidence that it is involved in the processes leading to bone loss in periodontitis (Sims et al., 2004). In addition, the levels of sTNF-R1, IL-10, and IL-19 also differed significantly between periodontitis patients and healthy individuals. The salivary levels of IL-19 has not previously been reported as elevated in patients with periodontitis, but we have previously reported that the gene *IL-19* is significantly up-regulated in gingival tissue biopsies from patients with periodontitis compared to healthy controls (Lundmark et al., 2015).

We further investigated the co-variance between microbiota and cytokines by employing a multivariate method, sPLS-DA. This analysis highlighted, for the first time, that gp130/sIL-6Rβ, the most significant in the DESeq2 analysis among cytokines, correlated positively with ASVs assigned to *Selenomonas sputigena, Selenomonas* sp., *Treponema* sp., *Streptococcus* sp. and an unclassified *Bacteria* (ASV471). Moreover, with regard to ASVs that had been identified in DESeq2 analysis or random forest model, ASVs assigned to *F. alocis, E. nodatum, Streptococcus* sp., and *Clostridiales* order were identified to negatively correlate with the anti-inflammatory cytokine IL-10. In addition, *G. elegans*, included in caries-associated taxa (Fakhruddin et al., 2019), positively correlated with IL-10. The above correlations between microbiota and expression of cytokines, identified for the first time here, may play an important role for the development and maintenance of the chronic inflammatory disease periodontitis. The combined profile of host-derived cytokines and microbes for prediction of dental diseases including periodontitis and caries has previously been suggested (Gursoy et al., 2011; Gursoy and Kononen, 2016; Belstrom et al., 2017a; Simon-Soro et al., 2018). With regard to periodontitis, a significant association between MMP-9, osteoactivin, IL-8, and macrophage inflammatory protein 1α and bacterial composition microbiota and cytokine profile in GCF has been identified in periodontal host homeostasis (Zhou et al., 2017).

Our sPLS-DA also revealed that the microbial data had a better discriminatory power between samples from patients and healthy controls compared to the cytokine dataset. A large number of studies have focused on identifying inflammatory mediators that could be used as biomarkers for periodontitis. However, our results indicate that the salivary microbiota has better discriminatory power between disease and health, than has the cytokines. Moreover, we present significant correlations between salivary microbiota and host-derived inflammatory mediators, suggesting that interactions might be a future approach to identify prognostic/diagnostic tools for the complex disease periodontitis.

One of the limitations with our study is the differences in age distribution in the two groups included in the study that might potentially affect the results. Additional limitations with this study, which may have impacted on results, include the lack of information on smoking habits and the gender differences between the periodontitis and healthy group (36/30 and 32/16 females/males, respectively). On the other hand, when comparing our salivary microbiota findings with previous studies, the ASVs we identified as most important in the samples from patients with periodontitis included the well-known periodontal pathogens such as *Porphyromonas gingivalis, Tannerella forsythia*, and *Fusobacterium nucleatum*. Moreover, the species *E. saphenum, T. forsythia, F. alocis*, and *P. micra*, which we identified as more abundant in samples from patients with periodontitis, compared to healthy controls, have previously been associated with periodontitis also in plaque samples as described above. Nonetheless, additional studies are needed to further investigate our findings on well age- and gender-matched participants with known smoking habits. The strengths of our study were that we used a relatively large sample size and periodontally well-characterized participants to investigate the combination of microbial and host biomarkers in a single study employing robust techniques.

Taken together, our findings reveal distinct and disease-specific patterns of salivary microbial composition of samples from patients with periodontitis compared to healthy controls, as well as significant correlations between microbiota and host inflammatory mediators. The positive correlations between the pathogens *Treponema* sp. and *Selenomas* sp. and the cytokines chitinase 3-like 1, sIL-6Rα, sTNF-R1, and gp130/sIL-6Rβ might have the future potential to serve as a combined bacteria-host salivary biomarker panel for diagnosis of the chronic infectious disease periodontitis. However, further studies are required to determine the capacity of these microbes and inflammatory mediators as a salivary biomarker panel for periodontitis.

## Data Availability

The datasets generated and analyzed during the current study are available in the European Nucleotide Archive (ENA) repository, under accession number PRJEB21767.

## Ethics Statement

This study was carried out in accordance with the recommendations of the Declaration of Helsinki and current Swedish legislation with written informed consent from all subjects. All subjects gave written informed consent in accordance with the Declaration of Helsinki. The protocol was approved by the Regional Ethics Board in Stockholm with reference number 2014/1588 – 32/3.

## Author Contributions

TY-L, AA, AL, and YH contributed to the study concept and design. GJ collected samples and obtained clinical metadata. AL and YH performed the experiments. AL, YH, and MH analyzed the data. AL, TY-L, and YH wrote the manuscript. All authors revised and approved the final version of the manuscript.

### Conflict of Interest Statement

The authors declare that the research was conducted in the absence of any commercial or financial relationships that could be construed as a potential conflict of interest.

## Supplementary Material

Supplementary Table S1 Taxonomic differences between saliva samples from patients with periodontitis and healthy controls, reported at phylum, class, order, family, and genus level. Taxa more abundant in saliva samples from patients with periodontitis are highlighted in red and taxa more abundant in healthy controls are highlighted in blue.

Supplementary Table S2 Significantly differentially abundant sequence variants in saliva samples from patients with periodontitis compared to healthy controls.

Supplementary Table S3 The 20 sequence variants with the highest variable importance scores in the random forest classification.
